# FAP-a and GOLPH3 Are Hallmarks of DCIS Progression to Invasive Breast Cancer

**DOI:** 10.3389/fonc.2019.01424

**Published:** 2019-12-17

**Authors:** Li-Na Yu, Zhen Liu, Yan Tian, Pei-Pei Zhao, Xing Hua

**Affiliations:** ^1^Department of Pathology, Nanfang Hospital, Guangzhou, China; ^2^Department of Pathology, School of Basic Medical Sciences, Southern Medical University, Guangzhou, China; ^3^Guangdong Provincial Key Laboratory of Molecular Tumor Pathology, Guangzhou, China; ^4^Department of Pathology, School of Clinical Medicine, Guizhou Medical University, Guiyang, China; ^5^Department of Pathology, Guangzhou Red Cross Hospital, Medical College, Jinan University, Guangzhou, China

**Keywords:** FAP-a, GOLPH3, DCIS, recurrence, breast cancer

## Abstract

Biological markers that could predict the progression of ductal carcinoma *in-situ* (DCIS) to invasive breast cancer (IDC) are required urgently for personalized therapy for patients diagnosed with DCIS. As stroma was invaded by malignant cells, perturbed stromal-epithelial interactions would bring about tissue remodeling. With the specific expression of the fibroblast activation protein-alpha (FAP-a), Carcinoma-associated fibroblasts (CAFs) are the main cell populations in the remodeled tumor stroma. Golgi phosphoprotein 3 (GOLPH3), a documented oncogene possessing potent transforming capacity, is not only up-regulated in many tumors but also an efficient indicator of poor prognosis and more malignant tumors. The present study aimed to retrospectively evaluate the pathological value of FAP-a and GOLPH3 in predicting the recurrence or progression of DCIS to invasive breast cancer. Immunohistochemical techniques were applied to investigate the expression of FAP-a GOLPH3 in 449 cases of DCIS patients received extensive resection and with close follow-up, but not being treated with any form of chemo- or radio-therapy. The combination of FAP-a and GOLPH3 in predicating the recurrence or progression of DCIS into invasive breast cancer was specifically examined. The study demonstrated that the overexpression of FAP-a in stromal fibroblasts and GOLPH3 in carcinoma cells are highly predictive of DCIS recurrence and progression into invasive breast cancer. Both FAP-a and GOLPH3 have high specificity and sensitivity to predict the recurrence of DCIS. Moreover, the combination of FAP-a and GOLPH3 tends to further improve the specificity and sensitivity of DCIS recurrence by 9.72–10.31 and 2.72–3.63%, respectively. FAP-a and GOLPH3 serve as novel markers in predicting the recurrence or progression of DCIS into invasive breast cancer.

## Introduction

With the development and aid of high quality screening mammography, the probability of detected ductal carcinoma *in situ* (DCIS) has raised rapidly (1). DCIS declares roughly 20% of all screening-detected breast cancer recently (2). Breast conserving therapy combined with or without radiotherapy has been generally accepted as the standard and efficient method to treat DCIS (3). Although DCIS is a type of non-invasive breast cancer, in which carcinoma cells are controlled by the basement membrane of breast duct, in reality, DCIS remains a heterogeneous cluster of pre-invasive breast cancers with various types of morphology, expansion, and malignant capacities (4).

DCIS remains the status of the carcinoma precursor lesion of invasive ductal carcinoma (IDC), but mechanisms under the conversion are largely unknown (5–7). In order to predict the fate of DCIS more accurately, to develop invasive cancer or remain at DCIS status, efforts must be made continuously to investigate the molecular aspects of the disease. In the last few years, several prognostic factors have emerged as possible contenders to help stratify risk (8). For example, a study by Martins et al. (9) demonstrated that monocarboxylate transporter 4 (MCT4) and stromal caveolin 1 (Cav-1) were differentially expressed in IDC and DCIS. Suppression of Cav-1 and activation of MCT4 in the stroma were indicators of overproduction of oxidative stress and glycolysis, indicating the progression from DCIS status into IDC.

The prolyl-specific serine proteinase fibroblast activation protein alpha (FAP-a), a type II integral membrane protein (10), is a M(r) 95,000 cell surface antigen brought about instantaneously by induced stromal fibroblasts during embryogenesis (11), which is the latter stages of wound healing (12), in some pathologic status where fibrous tissue growth is a conspicuous feature (13), and is occasionally present in normal fibroblast or pancreatic a-cells (14, 15). As shown in our previous studies, immunohistochemical analyses were applied to evaluate the expression of FAP-a in normal mammary tissues and usual ductal hyperplasia (UDH), DCIS, DCIS with microinvasive (DCIS-MI) and IDC. We further hypothesized the participation of FAP-a in designating the microemboli's formation, facilitating the pathological process of breast cancer (16). Cremasco et al. (17) showed that breast tumors contain a variety of FAP expressing stromal cells with dichotomy function, phenotype, and location. Busek et al. (18) also found that FAP is a promising therapeutic target. In this study, we found that FAP-a is specifically expressed in the stromal fibroblasts of IDC and the tumor-host interface of the invasive front of DCIS-MI. However, FAP-a is negatively expressed in stromal fibroblasts of normal mammary tissues and UDH. We are the first study that proposed and demonstrated FAP-a's potential as a novel biomarker for pathological diagnosis of DCIS with microinvasion. Although high FAP-a expression was detected in stromal fibroblasts of IDC and tumor-host interface at the invasive front of DCIS-MI, its correlation with the chemo- or radio-therapy treatment has not been studied because of the limited sample size.

Previous investigations have demonstrated that overexpression of human chromosome 5p13 contributed to tumor formation, progression, and metastasis. The expression of Golgi phosphoprotein 3(GOLPH3) was found to be correlated with copy number of 5p13 in human lung cancer, and gain or loss of function studies have proved that GOLPH3 was a genuine oncogene possessing strong transforming activity, which was induced in cancers with 5p amplification (19). Further researches on the clinical importance of GOLPH3 revealed that GOLPH3 expression was not only increased in oral tongue cancer and glioma, but also was indicating poor prediction and more malignant tumors (20–22). These results further indicated GOLPH3's significant role in the progression of various types of tumors. In our previous works, it was revealed that overexpression of GOLPH3 promoted the progression of prostate cancer from hormone sensitive stage to hormone stage, and GOLPH3's potential as a novel biomarker and potential target for antagonizing castration resistant prostate cancer (23). Zeng et al. showed that high expression of GOLPH3 was associated with low overall survival rate of breast cancer patients, and overexpression of GOLPH3 increased the proliferation and tumorigenicity of human breast cancer cells (24).

In the present study, immunohistochemical techniques were applied to evaluate the role of GOLPH3 in carcinoma cells and FAP-a in the stroma in recruited DCIS patients received combined treatment of wide-excision and close follow-up, but haven't received any type of chemo- or radio-therapy; which further explored the potentials of FAP-a and GOLPH3 as indicative marker during the natural progression of DCIS. To this end, we specifically examined the correlation between FAP-a and GOLPH3 expression, their clinico-pathological values in predicting the recurrence or progression of DCIS into invasive breast cancer.

## Materials and Methods

### Case Selection

We retrospectively recruited 449 patients who had received treatment of mastectomy between May 1994 and May 2013 using the surgical oncology breast cancer database. Archives were acquired from the documentation of the Department of Pathology and Medical Records Room, the Fourth Affiliated Hospital of Jinan University and Nan Fang Hospital of Southern Medical University in Guangzhou, China. This study was approved by the Ethics Committee of Guangzhou Red Cross Hospital.

Formalin-fixed and further paraffin-embedded (FFPE) tissue sections were prepared from 449 patients with DCIS. All patients undergoing surgical excision were treated by the same surgeon, and all had negative margins (= or > 10 mm) at the end of surgical excision or re-excision. None of the patients received radiation treatment or tamoxifen therapy. The time of diagnosis and recurrence was recorded as the time of surgery that initiated the pathologic diagnosis. Data analysis indicated the endpoint of treatment based on the appearance of local recurrence of DCIS, invasive breast carcinoma and without pathological symptoms at the last time of follow-up. As to each subject, histologic patterns (comedo, cribriform, micropapillary, papillary, or solid), presence of necrosis, inflammation and nuclear grade were kept on record. Routine morphological analysis of hematoxylin and eosin stained tissue sections were blindly reviewed by five pathologists together and a diagnostic agreement was finalized.

### Immunohistochemistry

The expression of GOLPH3 in tumor cells and FAP-a in tumor stroma were evaluated by immunohistochemical staining. Firstly, sections were deparaffinized by reduced xylene and then conducted antigen retrieval in Tris-EDTA retrieval buffer (PH 9.0) (Dako, Carpinteria, CA) before immunohistochemical staining with a Dako autostainer. Immunohistochemical staining for GOLPH3 and FAP-a was completed as described previously. The GOLPH3 primary antibodies (clone 19112-1-AP, 1:100 dilution) were obtained from the ProteinTech Group, and the FAP-a primary antibodies (clone 427819, 1:50 dilution) were obtained from R & D systems. Positive and negative staining were carried out for each staining. The degrees of staining were graded based on the following criteria. Grade 1 was taken as semi-quantitative and negative staining; Grade 2 was taken when either diffuse weak staining or <30% strong staining appeared; Graded 3 was awarded when 30% or more of the cells have been stained.

### Statistics

The time from surgery to recurrence (TTR) was recorded and evaluated by using Kaplan-Meier survival curves. The comparison of stratified survival curves used log-rank tests. Cox proportional hazards regression was applied to analyze the correlation among FAP-a, GOLPH3 expressions, and TTR with other potential diagnostic markers of TTR. All hypotheses testing was conducted using the Fisher exact test or the Kruskal-Wallis test, based on the discrete or continuous essence of any other factor. Correlation analysis among FAP-a, GOLPH3 staining intensity, and DCIS recurrence rate was determined using the Spearman test.

*P*-values were all two-sided, and *p* < 0.05 was considered as statistical significance. Statistical analysis was accomplished using the SPSS statistical analysis software version 19.0, and tables and graphs were generated.

## Results

### Informative Analysis of the DCIS Patient Cohort

**Figure 1:**
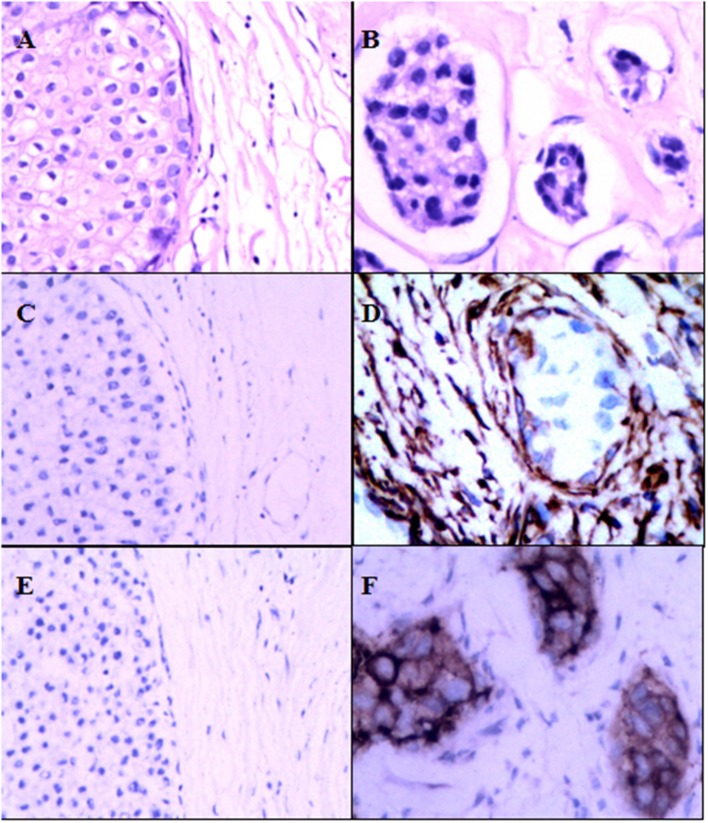
Expression of FAP-a and GOLPH3 in DCIS and IDC. **(A)** Hematoxylin and eosin (H & E) staining of DCIS. **(B)** Hematoxylin and eosin (H & E) staining of IDC. **(C)** IHC staining of FAP-a in DCIS. **(D)** IHC staining of FAP-a in IDC. **(E)** IHC staining of GOLPH3 in DCIS. **(F)** IHC staining of GOLPH3 in IDC.

As shown in Table 1, 449 women were with a median age of 52 years (range 22–87 years) when diagnosed with DCIS. The median of follow-up time was 169.84 months (14.15 years). Pathological variables including grade, histologic pattern and necrosis were recorded accordingly. Meanwhile, follow-up on DCIS recurrence pattern and DCIS deterioration into invasive breast cancer were also recorded. The immunophenotype of GOLPH3 and FAP-a is correlated with each clinicopathological parameter listed in Table 1. As shown in Figure 1, the FAP-a protein expression was mainly detected in the cytoplasmic fractions of stromal fibroblasts, while GOLPH3 in was detected the cytoplasm of breast carcinoma cells. In these 449 DCIS patients, 110 of which appeared certain degree of recurrence (49 progression into DCIS, while the other 61 progression into invasive breast cancer), resulting in an final recurrence rate of 24.50%. Therefore, the progressive rate of DCIS into invasive breast cancer at the present cohort study was 13.59 percent (61/449), which is relevant to the expected rate of 12–15%. The median time of recurrence turned out to be 83.25 months (DCIS with 33.45 months, invasive breast cancer with 115.36 months).

**Table 1:** Levels of FAP-a and GOLPH3 expression in relation to clinicopathologic variables.

**Characteristics**	**FAP-a expression**		**GOLPH3 expression**	
	**Negative**	**Positive**	**Negative**	**Positive**
Number	316	133	321	128
**Age, year**				
≤50	137	58	145	49
>50	179	75	176	79
**Grade**				
Low	88	29	47	21
Moderate	107	33	176	69
High	121	71	98	38
**Histological pattern**				
Solid	58	28	62	27
Cribriform	121	32	116	23
Papillary	11	8	15	11
Micropapillary	29	7	27	18
Comedo	97	58	101	49
**Necrosis**				
Yes	135	51	145	77
No	181	82	176	51

### Stromal FAP-a Is Associated With DCIS Recurrence

**Figure 2:**
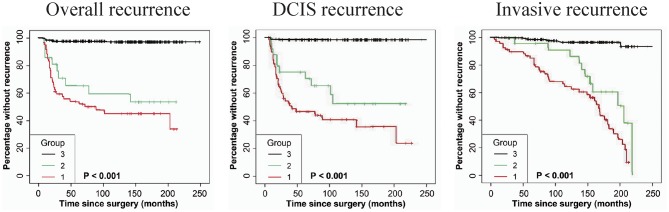
Kaplan-Meier curves for stromal FAP-a state and duration to recurrence among DCIS patients. **(Left panel)** The expression or overexpression of stromal FAP-a is correlated with the elevation of overall recurrence. **(Middle panel)** The expression or overexpression of stromal FAP-a is correlated with the elevation of DCIS recurrence. **(Right panel)** The expression or overexpression of stromal FAP-a is positively correlated with the increase of DCIS progression to IDC. *p*-values (log rank test) are as shown.

As shown in Table 2, immunostaining of stromal FAP-a was scored semiquantitative as 3 (high), 2 (low), and 1 (absent of staining) based on the staining intensity. In 49 DCIS recurrence cases, the percentages of grade 3, 2, and 1 of stromal FAP-a immunostaining were 65.31% (32/49), 18.37% (9/49), and 16.33% (8/49), respectively. In 61 DCIS patients who recurred to IDC, percentages of grade 3, 2, and 1 of FAP-a immunostaining were 65.57% (40/61), 19.67% (12/61), and 14.75% (9/61), respectively. In 339 DCIS patients without recurrence, the incidence of high, low, and absent stromal FAP-a immunostaining was 7.96% (27/339), 3.54% (12/339), and 88.50% (300/339), respectively. Spearman test showed no significant correlation between FAP-a staining intensity and DCIS recurrence in DCIS recurrence patients. Among 132 FAP-a positive DCIS patients, 41 recurred to DCIS, 52 recurred, and progressed into invasive breast cancer, while the remaining 39 cases experienced no recurrence. FAP-a (high/low) is expressed in 84.55% (93/110) of the DCIS recurrence patients, while only 11.50% (39/339) in DCIS patients without recurrence. This difference was statistically significant (*P* < 0.001). By contrast, no statistical difference was detected of FAP-a expression between DCIS recurrence and invasive progression. Kaplan-Meier curves for overall recurrent rate, rate of recurrent to DCIS, and progression into invasive breast cancer data were revealed in Figure 2. The left panel suggested that for the patients with higher expression of FAP-a, the overall recurrent rate will be higher. The middle panel suggested that the higher expression of FAP-a may be correlate with the higher rate of recurrent to DCIS. The right panel suggested that the patients with higher expression of FAP-a may have the higher possibility of progression to invasive breast cancer. It was worth noting that the presence of stromal FAP-a was positively correlated with DCIS recurrence.

**Table 2:** The expression of stromal FAP-a is associated with DCIS recurrence.

	**Stromal FAP-a status**						
**Recurrence type**	**Total cases**	**Score** **=** **3**		**Score** **=** **2**		**Score** **=** **1**	
		***N***	**%**	***N***	**%**	***N***	**%**
None	339	27	7.96	12	3.54	300	88.50
DCIS	49	32	65.31	9	18.37	8	16.33
IDC	61	40	65.57	12	19.67	9	14.75
Overall (DCIS + IDC)	110	72	65.45	21	19.09	17	15.45

### GOLPH3 Expression Is Associated With DCIS Recurrence

**Figure 3:**
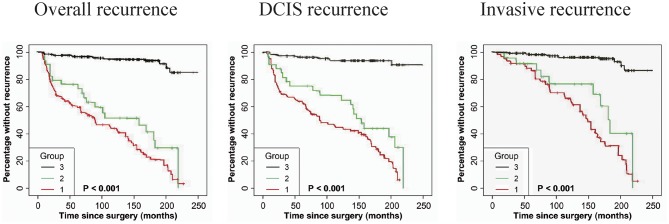
Kaplan-Meier curves for GOLPH3 state and duration of recurrence among DCIS patients. **(Left panel)** The expression of GOLPH3 is positively associated with the induction of overall recurrence. **(Middle panel)** The expression of GOLPH3 is correlated with the increase of DCIS recurrence. **(Right panel)** The expression of GOLPH3 is associated with the induction in DCIS progression to IDC. High (score = 3), low (score = 2), and absent (score = 1). *p*-values (log rank test) are as shown.

As shown in Table 3, immunostaining of stromal GOLPH3 was scored semiquantitative as 3 (high), 2 (low), and 1 (absent of staining) based on the staining intensity. In 49 DCIS recurrence cases, the incidence of high, low, and absent GOLPH3 immunostaining was 69.39% (34/49), 20.41% (10/49), and 10.20% (5/49), respectively. In 61 DCIS patients who recurred to IDC, the incidence of high, low, and absent GOLPH3 immunostaining was 62.30% (38/61), 16.39% (10/61), and 21.31% (13/61), respectively. In the 339 DCIS cases without recurrence, the percentages of grades 3 (high), 2 (low), and 1 of GOLPH3 immunostaining were 6.78% (23/339), 4.13% (14/339), and 89.09% (302/339), respectively. Spearman test showed no significant correlation between the GOLPH3 staining intensity and DCIS recurrence in DCIS recurrence patients. Among the 132 GOLPH3 positive DCIS patients, 44 of them underwent a recurrence to DCIS, 48 recurred and progressed into invasive breast cancer, while the remaining 37 cases did not experienced no recurrence. GOLPH3 (high/low) is expressed in 83.64% (92/110) of the DCIS recurrence patients while in 10.91% (37/339) of the DCIS without recurrence. This difference was statistically significant (*P* < 0.0001). By contrast, no statistical difference was detected regard of GOLPH3 expression between DCIS recurrence and the invasive progression of DCIS. Kaplan-Meier curves for overall recurrence, DCIS recurrence, and progression into invasive breast cancer were shown in Figure 3. Note that the presence of GOLPH3 is specifically associated with DCIS recurrence.

**Table 3:** GOLPH3 expression is associated with DCIS recurrence.

	**GOLPH3 status**						
**Recurrence type**	**Total cases**	**Score** **=** **3**		**Score** **=** **2**		**Score** **=** **1**	
		***N***	**%**	***N***	**%**	***N***	**%**
None	339	23	6.78	14	4.13	302	89.09
DCIS	49	34	69.39	10	20.41	5	10.20
IDC	61	38	62.30	10	16.39	13	21.31
Overall (DCIS + IDC)	110	72	65.46	20	18.18	18	16.36

### The Combination of FAP-a and GOLPH3 Expression Is Associated With DCIS Recurrence

**Figure 4:**
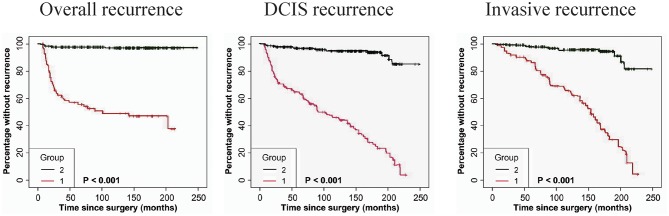
Kaplan-Meier curves for FAP-a and GOLPH3 state and duration for recurrence among DCIS patients. **(Left panel)** The FAP-a^+^GOLPH3^+^ immunophenotype is correlated with the induction of overall recurrence. **(Middle panel)** The FAP-a^+^GOLPH3^+^ immunophenotype is associated with the increase of DCIS recurrence. **(Right panel)** The FAP-a^+^GOLPH3^+^ immunophenotype is correlated with the induction of DCIS progression to IDC. (FAP-a^+^GOLPH3^+^ immunophenotype, score = 1; FAP-a^−^GOLPH3^−^, score = 2). *p*-values (log rank test) are as shown.

The immunostaining of FAP-a in stromal fibroblasts and GOLPH3 in carcinoma cells was scored semiquantitatively as 3 (high), 2 (low), and 1 (absent of staining) based on the staining intensity, respectively. The staining intensity 2 and 3 were considered as positive and the staining intensity 1 was considered as negative. As shown in Table 4, 41 of the 49 DCIS recurrence patients showed both FAP-a positive in stromal fibroblasts and GOLPH3 positive in carcinoma cells (FAP-a^+^GOLPH3^+^), three of them showed FAP-a^−^GOLPH3^+^, five showed FAP-a^−^GOLPH3^−^, while none of them showed FAP-a^+^GOLPH3^−^. Among the 61 DCIS patients who recurred to IDC, 48 showed FAP-a^+^GOLPH3^+^, four showed FAP-a^+^GOLPH3^−^, nine showed FAP-a^−^GOLPH3^−^, while none of them showed FAP-a^−^GOLPH3^+^. Moreover, FAP-a^+^GOLPH3^−^ existed in 80.91% (89/110) of DCIS recurrence patients, while only 1.18% (4/339) of DCIS patients without recurrence (4/339) showed FAP-a^+^GOLPH3^+^ with statistically significant difference (*P* < 0.0001). Pearson test demonstrated a positively correlative relationship among FAP-a, GOLPH3 co-expression and DCIS recurrence. As shown Figure 4, Kaplan-Meier curves were revealed for overall recurrence, DCIS recurrence, and invasive recurrence progressing into invasive breast cancer. Note that the co-expression of FAP-a and GOLPH3 are specifically associated with DCIS recurrence.

**Table 4:** Specificity and sensitivity of FAP-a and GOLPH3 in diagnosis of DCIS recurrence.

		**FAP-a and GOLPH3 status**							
**Recurrence type**	**Cases**	**FAP-a^+^**GOLPH3**^+^**		**FAP-a^−^**GOLPH3**^+^**		**FAP-a^+^**GOLPH3**^−^**		**FAP-a^−^**GOLPH3**^−^**	
		***N***	**%**	***N***	**%**	***N***	**%**	***N***	**%**
None	339	4	1.18	20	5.90	38	11.21	277	81.71
DCIS	49	41	83.67	3	6.12	0	0	5	10.21
IDC	61	48	78.69	0	0	4	6.56	9	14.75
Overall (DCIS+IDC)	110	89	80.91	3	2.73	4	3.64	14	12.73

### Specificity and Sensitivity of FAP-a and GOLPH3 in Diagnosis of DCIS Recurrence

The immunostaining of FAP-a in stromal fibroblasts and GOLPH3 in carcinoma cells was scored semiquantitatively as 3 (high), 2 (low), and 1 (absent of staining) based on the staining intensity, respectively. The staining intensity 2 and 3 were considered as positive and the staining intensity 1 was considered as negative. As shown in Table 5, the specificity and sensitivity of FAP-a in the diagnosis of DCIS recurrence is 88.50 and 84.55%, respectively. The specificity and sensitivity of GOLPH3 in the diagnosis of DCIS recurrence is 89.09 and 83.64%, respectively. When combined FAP-a and GOLPH3, the specificity and sensitivity in diagnosis of DCIS recurrence increased to 98.82 and 87.27%, respectively.

**Table 5:** Association of the FAP-a and GOLPH3 with DCIS recurrence.

	**Specificity**		**Sensitivity**	
	***N***	**%**	***N***	**%**
FAP-a	300/339	88.50	93/110	84.55
GOLPH3	302/339	89.09	92/110	83.64
FAP-a + GOLPH3	335/339	98.82	96/110	87.27

## Discussion

DCIS is considered as a precursor to invasive breast cancer. In this study, we demonstrated that 24.50% of DCIS patients undergo an ipsilateral local recurrence, and 13.59% of these recurrences are invasive in a sample of 449 DCIS patients. The risk of DCIS patients of local recurrence appearance or progression into invasive breast cancer after their treatment was still unpredicted at the present stage (25). The ability to develop a predictive model would present to be an enormous clinical advance to identify a molecular marker that would have predictive potential of DCIS's prognosis, either DCIS develops invasive cancer or stays DCIS.

Although several molecular factors have emerged as predictive marker of DCIS in the last few years, it remains challenging to clarify the prognostic significance of those bio-markers. In most studies, sample sizes were limited, and patients had undergone either endocrine treatment or radiotherapy (8, 9). In this study, all of the patients undergone only surgical excision and were treated by the same surgeon, and all of them are with negative margins to the end of surgical excision. None of the patients received radiation therapy or tamoxifen treatments, which potentiates us to evaluate the predictive potential of FAP-a and GOLPH3 during the natural history of DCIS disease progression.

Tissue remodeling stimulated by the interactions of perturbed stromal-epithelial cells is brought about during the carcinogenesis of DCIS recurrence (16). Although these interactions turns to of great importance in tumorigenesis, their underlying molecular mechanisms in tumorigenic processes are not fully understood. Most studies demonstrated that carcinoma-associated fibroblasts (CAFs) remain the major stromal cell population participating in the epithelial-stromal interaction in the remodeled micro environment, which were mainly responsible for production of FAP-a (5, 26, 27). In this study, 84.55% of the DCIS recurrence showed high/low expression of FAP-a expression in stromal fibroblasts, while only 11.50% of the DCIS without recurrence showed high/low FAP-a expression. Significant difference in the protein expression of FAP-a was detected between the DCIS recurrence group and without recurrence group. We have previously demonstrated that 82.72% (67/81) stromal fibroblasts in the invasive front of DCIS with microinvasion at the tumor-host interface showed significant FAP-a positivity, and all of the 67 cases of IDC exhibited strongly positive FAP-a staining in stromal fibroblasts. Thus, FAP-a was potentiated in the promotion of the formation of microemboli, which promotes the invasion of breast cancer. Hence, the micro environmental reprogramming of cancer revealed as a key step in DCIS recurrence, and it may of greater importance to elucidate stromal metabolism other than characteristic of carcinoma cells to predict the prognosis of DCIS patients by characterizing.

Interestingly, our study elucidated that the presence of GOLPH3 in carcinoma cells is also specifically associated with DCIS recurrence. In this study, 83.64% of the DCIS recurrences demonstrated high/low GOLPH3 expression in carcinoma cells, while only 10.91% of the DCIS without recurrence showed high/low GOLPH3 expression. In addition, a significant difference was found in the expression of GOLPH3 protein between the DCIS recurrence group and without recurrence group. Several studies have shown that GOLPH3 is related to the prognosis of cancer. It has been reported that high GOLPH3 expression is associated with poor overall survival in patients with breast cancer and that GOLPH3 overexpression increases the proliferation and tumorigenicity of human breast cancer cells (24). In prostate cancer, patients with high levels of GOLPH3 will have shorter survival time (28). Zhang et al. (29) has found that GOLPH3, which is up-regulated in prostate cancer tissues, can be useful for predicting biochemical recurrence-free survival and overall survival in patients with prostate cancer. Moreover, GOLPH3 may be of significant potential as predictive marker of DFS and OS in subjects with diagnosed prostate cancer (23). Not only prostate cancer, but also GOLPH3 has been proved to be highly expressed in NSCLC tissues, indicating that GOLPH3 may be a useful diagnostic factor for NSCLC (30). Tang et al. found that high expression of GOLPH3 usually indicates poor survival of breast cancer and weak resistance to chemotherapy (31). GOLPH3L is an accessory homolog of GOLPH3. It had been found that the prognosis of ovarian cancer patients with high expression of GOLPH3L was lower than that of patients with low or no expression of GOLPH3L (32).

Meanwhile, the detection of GOLPH3 expression might serve as a reliable predictive marker. Same is reasonable that to determine the changes of GOLPH3 expression may also facilitate to comprehensively understand the incidence of progression in patients with prostate cancer.

To our understanding, a group of proteins constitutes in the trans-Golgi matrix were tightly regulated in the trans-Golgi and demonstrated to be responsible for anterograde and retrograde Golgi trafficking, as well as collaborating with the cytoskeleton and supporting the Golgi structure (33). GOLPH3 would transiently be transported to the surface of cytoplasm in the trans-Golgi and represented a first-in-class Golgi oncoprotein. Holly C. Dippold indicated that trafficking out of the Golgi relied on the interaction of GOLPH3, MYO18A, F-actin, and PtdIns (4) P. This process is controlled by MYO18A and F-actin and their transmission to the Golgi regulated by GOLPH3 and PtdIns (4) P. This evidence suggests that GOLPH3 traffics the tensile force responsible for successful tubule and vesicle formation (20). Kenneth L. Scott raised the hypothesis that GOLPH3 interacted with VPS35 and the tetramer to recycle receptor for key molecules, thus regulating downstream mTOR signaling (19). Tenorio et al. found that the cytosolic and membrane components of the three breast cell lines had biochemical differences in GOLPH3, that was that, in cancer cells, part of the overexpressed GOLPH3 was modified by differentiation (34).

We have shown here that the presence of FAP-a in stromal fibroblasts and GOLPH3 in carcinoma cells are reliable biomarker of DCIS recurrence and progression into invasive breast cancer. Both FAP-a and GOLPH3 have high specificity and sensitivity in predicting the recurrence of DCIS. The specificity and sensitivity of FAP-a in diagnosis of DCIS recurrence was 88.50 and 84.55%, respectively. The specificity and sensitivity of GOLPH3 in the diagnosis of DCIS recurrence was 89.09 and 83.64%, respectively. However, the combination of FAP-a and GOLPH3 had higher specificity and sensitivity that the specificity and sensitivity was 98.82 and 87.27%, respectively. Compared with FAP-a and GOLPH3, the combination of FAP-a and GOLPH3 refined the specificity and sensitivity of potent prediction of DCIS recurrence by 9.72–10.31 and 2.72–3.63%, respectively. Thus, we identified that FAP-a and GOLPH3 might potentiate as a reliable biomarker for predicting the DCIS recurrence and progression of DCIS into invasive breast cancer. Though FAP-a and GOLPH3 showed significant correlation both in DCIS recurrence and in DCIS without recurrence patients, the complex interplay between FAP-a and GOLPH3 in DCIS recurrence remains poorly understood.

## Data Availability Statement

The datasets generated for this study are available on request to the corresponding author.

## Author Contributions

XH and L-NY: study concepts. L-NY, ZL, YT, and P-PZ: study design and statistical analysis. YT, P-PZ, and ZL: data acquisition. L-NY: quality control of data and algorithms. L-NY and ZL: data analysis and interpretation. XH: manuscript preparation. ZL and L-NY: manuscript editing. XH, L-NY, ZL, and P-PZ: manuscript review.

### Conflict of Interest

The authors declare that the research was conducted in the absence of any commercial or financial relationships that could be construed as a potential conflict of interest.
